# Medico-Legal Issues in Cremation: Comparative Analysis of International Legislation

**DOI:** 10.3390/healthcare10122428

**Published:** 2022-12-01

**Authors:** Pierpaolo Di Lorenzo, Gaetano Di Donna, Ludovica Busillo, Maria Pieri, Emanuele Capasso, Fabio Policino, Claudia Casella, Massimo Niola

**Affiliations:** Department of Advanced Biomedical Science-Legal Medicine Section, University of Naples “Federico II”, 80131 Naples, Italy

**Keywords:** cremation, body, burn, death, consent

## Abstract

Cremation has seen a constant increase in popularity all around the world. Because of its extensively destructive nature, however, a series of medico-legal issues arise concerning identification, forensic autopsy, external examination, histological, toxicological and genetic exams to be performed not in the immediacy of death. The aim of this study is to compare the international legislation on cremation, seeking the response of various countries to their medico-legal issues. Several affinities but also some differences were found. Similarities include the need for a certificate by a medical examiner excluding any medico-legal issues and non-natural causes of death and the expression of consent to cremation given by the deceased when still alive otherwise by relatives. Significant differences were found in German law, which provides for a second medical examination prior to cremation and in Italian law providing for the medical examiner to collect biological samples from the body and store them for a minimum of ten years for any future purpose of justice. The Italian approach could give a plausible solution to the medico-legal issues raised by cremation with the imperative premise, however, we need to look deeply into its privacy and consent implications, cost–benefits rate, sample collection and storage protocol.

## 1. Introduction

Cremation is a well-established mode of disposal, whose popularity has constantly increased all around the world [1,2], with an additional rise determined by the need for a rapid method of disposal of bodies during the COVID-19 pandemic [3,4,5].

Nations with the highest cremation rates (80% or higher) include Japan (over 99%), Hong Kong, Republic of Korea, India, Switzerland, Sweden, Czech Republic, Singapore, and Thailand [6,7].

Italy has seen an increase in cremation rates up to 27.31% in 2020 in comparison to 2019, for a total amount of 277.106 cremations in 2020 [8] in comparison to 232.974 of 2019.

The cremation process takes place in a specially designed furnace (cremation chamber) where the body is exposed to extreme temperatures (among 900–1000 °C), which leads to complete incineration. Remains are then usually further destroyed by a special processor into the final resulting ashes and, depending on the body size and weight, the type of coffin, and the efficiency of the equipment installed, the procedure usually takes between 1 and 2 h [9].

Due to its extensively destructive nature, cremation poses several medico-legal issues with critical aspects mainly concerning the correct identification of the urn content [10]. DNA analyses of ashes and bone fragments have been proved to be highly unreliable [11,12,13,14] because of both the acute and significant DNA degradation at high temperatures and the frequent occurrence of commingling of the remains [15]. Promising but still limited results in the DNA analysis of degraded petrous bones have been highlighted in a 2019 study focused on such specific matrix and aimed to solve forensic cases [16].

Moreover, the opportunity to perform (or to repeat) a forensic autopsy or histological, toxicological and genetic exams not in the immediacy of death is permanently lost.

Said challenges were exacerbated by the COVID-19 pandemic as numerous countries, especially in the first months of global spread of the disease, had significantly limited chances to perform autopsies [17], while ethical dilemmas related to consent collection and overruling of religious beliefs quickly arose [18].

These issues, well-known in the international literature, still lack fully satisfying answers: Katsos et al. [19] highlighted the challenges of identification and risk of deaths caused by violent agents not being adequately recognized, also identifying significant ambiguities in the Greek legislative framework and the role of coroners, while Radu et al. [20] posed questions related to the cremation of bodies subject to forensic expertise or that raised/may raise suspicions about the cause of death.

Additionally, controversy still exists around the efficiency and need of post-mortem external examinations prior to cremation, as Gill et al. [21] found no statistically significant difference between a physical inspection and medico-legal investigations without inspections in detecting unnatural unreported deaths. Consequently, the work effort and costs of performing physical inspections appears unjustified [21] while, on the other hand, deficiencies are known in the thoroughness and accuracy of the medical certificates for cremation [22,23]. Behrens et al. [24] and Heide et al. [25] found significant shortcomings in the initial post-mortem external examination, so that a second examination at the crematory (carried out by a medical professional specialized in this field) provides additional benefits, suggesting its importance for quality assurance of external examinations.

In this climate of uncertainty, the aim of this study is to compare the international legislation on cremation, looking for similarities and differences in the approach of different countries to the subject, seeking their response to these medico-legal issues.

## 2. Materials and Methods

By using the Google Chrome search engine with the keywords “legge cremazione”, “cremation law”, “feuerbestattungsgesetz”, “loi sur la crémation”, “ley de cremación”, “lei de cremação”, “kremasjonsloven”, “cremation law United States” and “cremation law Japan”, the cremation laws, respectively, of Italy [26], the United Kingdom [27], Germany (Berlin) [28], France [29], Spain (Castilla y Leon) [30], Portugal [31], Norway [32], the United States of America (New York [33] and Florida [34]) and Japan [35] were analyzed with respect to the latest official text published on governmental websites of these laws. Any overlapping and/or differences between national legislations were highlighted and commented.

## 3. Results

Italian cremation Law no. 130 “Cremation and ashes dispersal provisions” issued in 2001 provides that the authorization for cremation is given by a civil status officer of the municipality relevant to the death. To proceed with cremation, the decedent must have expressed his/her consensus during life (in a will or in free form handwritten writings) or must have communicated it verbally to relatives who, after death, will declare this wish in the manner defined by the relevant legislation.

More importantly, to be authorized, a certificate in free paper from the medical examiner excluding suspicion of death due to a criminal offence is required or, in the event of a sudden or suspicious death reported to the judicial authority, cremation must be authorized by the Prosecutor Office. Lastly, this law introduced the obligation for the medical examiner to collect samples of biological fluids and skin annexations from the body and store them for a minimum period of ten years for any future purpose of justice. In daily practice, this law has still not been widely applied, because it defers to a ministerial regulation that has never been issued. The Minister of Health delegated the issuance of the implementing decree to local regions that must organize the activity in all its aspects from the service fee to the sample storage. Italian regions, for their part, ulteriorly delegated this job to the single local health authorities (ASLs), which never produced a unified protocol, complying all aspects, with critical differences in procedures adopted between different regions of the country. For example, in 2018 in Abruzzo [36,37], the regional council reiterated to the ASLs the task, yet not completed, of ensuring the adequate number of specialized and adequately trained staff needed to carry out the biological samples collection.

In the Region of Sardegna (ASSL of Carbonia [38,39]), instead, an operating procedure was elaborated in 2017: a skin flap including hairs is collected with a scalpel and directly stored in formalin (no specific body area is specified); the same area is, then, used as a source for blood or exudate fluid collection, wiped with the use of filter paper (volume to be sampled is not specified). These guidelines comprehend pacemaker removal and the collection of deceased documentation, including cremation request, to be forwarded together with the samples to the Local Health Authority.

In addition, Campania [40] produced precise guidelines in 2017: samples of buccal mucosa are collected through a dry oral swab that is rubbed on the inside of the cheek, and samples are then transferred on appropriate cards. Each biological sample must be placed in a separate container consisting of an burglar-proof envelope with barcode identification. The removal of hair appendages (hair, axillary or pubic hairs) should be carried out with attention to the bulbs. If said samples are not available, vitreous humor should be sampled from the right or left lateral chant of the eye with a sterile syringe: the needle must penetrate a few millimeters into the chamber of the eye to aspirate the liquid (volume is not specified), which will be placed on a special card and stored in the same way as the buccal pad. The medical examiner will take blood fluid directly from the cavities with a syringe or, in cases of technical difficulty, with a femoral syringe or on the neck vessels; the blood sample will then be placed in EDTA tubes and transferred on chromatographic paper within 3 h. Said samples will be inserted, together with the cremation request, in a single sealed envelope burglar-proof and marked with a special code (whose nature is not clarified). Then, the package record will be computerized, allowing the traceability of operations to guarantee data protection. The procedure also specifies: “The barcode on the package containing the samples will be signed by the sampler with annotation regarding time of collection in the company register, together with serial number, barcode, date and time of collection, medical and personal data of the deceased”. Then, all will be managed informatically and exclusively accessible to authorized operators through a procedure of authentication and authorization. At the end of the 10-year storage period, the samples will be destroyed with the methods routinely used for biological materials, taking care to ensure the impossibility of associating the deceased to the destroyed sample.

In the United Kingdom, the law “The Cremation (England and Wales) Regulations 2008” as modified by two amendments in 2022 establishes that the request for cremation can be submitted by an executor of the deceased person or a near relative who has attained the age of 16 or by any other person if the medical referee is satisfied that the person is proper to make the application. Then, to obtain an authorization by the cremation authority, a certificate is given after external (or judicial) examination provided by the competent authority, according to the 2004 Act [41]. If post-mortem examination states the cause of death, the medical referee may authorize cremation. In some cases, a coroner’s certificate will be needed. These include cases when a post-mortem examination has been made and the cause of death has been certified by the coroner who states that no further investigations are needed. Authorization is requested also if an investigation has begun, the death occurred outside the British Islands and no post-mortem examination or investigation is necessary.

In Germany, any federal state has its own legislation about cremation. As an example, in Berlin, a body may be cremated only if a doctor from the competent forensic medical institute has established, after a second post-mortem examination, that the deceased died of natural causes. As a rule, the second post-mortem examination shall take place in the crematorium carrying out the cremation. If a natural cause of death cannot be reliably determined during the post-mortem examination, the doctor must provide further information and, if it is insufficient to determine a natural cause of death or if the post-mortem examination indicate that the deceased died of a non-natural cause, must notify the police authority without delay. Therefore, the prosecutor releases a body for cremation if it is no longer needed for evidence in the criminal case. This release is intended to ensure that cremation does not take place before the cause of death has been clarified in a way that excludes the liability of third parties.

In France, as provided by the article R2213-34 of the Code général des collectivités territoriales [28], cremation is authorized by the Mayor of the Municipality where death occurred or, if the body has been transported before being placed in a coffin, by the place where the coffin was closed. A written expression of the deceased’s last wishes is required or a request of any person who is qualified to provide for the funeral and provides proof of his marital status and domicile. To receive the authorization, then, a death certificate drawn up by the doctor certifying the death and stating that it does not pose any medico-legal problem is needed. In case of medico-legal problems, cremation can only take place after the authorization of the Prosecutor Office, who can order for an autopsy to be carried out by a forensic doctor chosen from the list of experts and at the expense of the family.

Similarly to Germany, Spanish legislation is different in each autonomous community. In the “Comunitat Autonoma di Castilla y Leon”, for example, as stated by article 18 of the “Decree 16/2005 of 10 February 2005 regulating the mortuary health police in the Community of Castile and Leon”, cremation is possible only when a medical certificate of death is available and the death registration certificate is issued by the Civil Registry Office. The cremation cannot be carried out before twenty-four hours after death nor after forty-eight hours after death, except:

- When an autopsy has been performed or tissues, organs or anatomical pieces have been obtained for transplantation, as appropriate, the corpse may be buried or cremated before twenty-four hours have elapsed after death.
- When the use of temporary preservation or embalming techniques is mandatory.

In Portugal, according to the law No. 411/98, no corpse may be buried, cremated, enclosed in a zinc coffin or placed in a cold chamber without the respective death certificate or death certificate having been previously drawn up. A body must be buried or cremated within the following maximum time limits:

- Within seventy-two hours, if immediately after the determination of death the body was delivered to the executor (alternatively to a spouse, cohabitee or any heir or relative);
- Within seventy-two hours from the time of entry into the national territory if it was transported from a foreign country to Portugal;
- Within forty-eight hours from the end of the medico-legal or clinical autopsy;
- Within twenty-four hours from the time it is delivered to one of the persons in case there is no medico-legal autopsy and there is danger to public health.

In addition, a certificate or declaration of death signed by two doctors or a coroner is required, together with the authorization for cremation signed by the deceased when alive or their legal representative.

In Norway, as stated by the “Act on cemeteries, cremation and burial (Cemetery Act)”, cremation must take place in an approved crematorium within ten working days. Requests for cremation are made by the person who arranges for the deceased’s funeral. At least three days before cremation, the crematorium must notify the police, who may ask for a delay in cremation if there are reasons to believe that a forensic autopsy is deemed appropriate.

In the United States of America, much like in Germany and Spain, legislation on cremations differs among the states.

As an example, in the State of New York, the funeral director contacts the next of kin to obtain personal and demographic information about the deceased person. The funeral director then sends the death certificate to the attending physician to certify the cause of death. If the death was caused by other than natural causes, the case is referred to the coroner or medical examiner to complete the medical certification.

A proper cremation permit must be issued by the medical examiner, and a “cremation authorization form” must be completed and signed by the legal next of kin before the crematory can accept remains for cremation.

In Florida, the legally authorized person contracting for cremation services shall be required to designate her or his intentions in a signed declaration of intent which shall be provided by and retained by the funeral or direct disposal establishment. When a body is to be cremated the medical examiner of the district in which the death occurred or the body was found must determine the cause of death, performing all examinations, investigations and eventually autopsy, they can proceed also after state attorney request. If there is no evidence of a violent or non-natural cause of death, the medical examiner signs a burial-transit permit, therefore authorizing cremation.

The cremation process may occur only after a period of 48 h and if the deceased person’s primary or attending physician or the medical examiner completes the medical certification of the death certificate within 72 h of receiving the death certificate from the funeral director. This person lists the cause of death on the certificate.

Finally, in Japan, the “Act on Cemetery, burial, etc. (Act No. 48 of 31 May 1948)” provides that cremation shall not be carried out until 24 h after death and that a permission must be obtained from the mayor of a municipality pursuant to the provisions of an Ordinance of the Ministry of Health, Labor and Welfare. Said permission shall be granted upon receipt of notification of death or stillbirth.

The study and comparison of international legislations on cremation showed many similarities regarding the need of a certificate by a medical examiner that excludes any medico-legal issues and non-natural causes of death and the requirements of a consent to cremation expressed by the deceased person when still alive or by relatives who, after death, will express such a wish.

Significant differences were also found, with regard to German legislation which provides for a second medical examination prior to cremation and Italian obligation for the medical examiner to collect samples of biological fluids and skin annexations from the body and store them for a minimum period of ten years (Table 1).

## 4. Discussion

The worldwide increase in cremation rates has its roots in different practical, ethical and religious issues: overcrowding of cemeteries, strategic planning issues related to the supply of cemetery and crematoria facilities, conservation of historic cemeteries [43] rise in prices of increasingly reduced cemeterial space, non-polluting nature of the practice and clearance by the Catholic Church [44].

As cremation gains popularity in all areas of the world, its extensively destructive nature and the subsequent medico-legal issues (Table 2) become progressively more important and should be adequately addressed.

The chances of investigating both the time and circumstances of death are significantly reduced, as the lack of biological samples suitable for forensic studies, which is usually guaranteed in cases of inhumated bodies by the presence of, at least, skeletal material [45], makes it impossible to pursue any future analysis, nonetheless of forensic relevance.

As a matter of fact, the practice of exhumation is often involved in forensic cases as it can be a great source of essential information [46,47] regarding the presence of macroscopic bone lesions and/or signs of violent death [48], as well as a valid substrate for toxicological [49,50,51] and genetic exams [52,53], even after significant time from death.

Cremation, instead, dramatically reduces the opportunity to find any macroscopical external and parenchymatous lesions or fractures [54,55], limiting the forensic studies to the immediacy of death, even though Franceschetti et al. [56] have highlighted the ever-present importance of an accurate analysis of burnt human remains for peri-mortem trauma identification, particularly concerning head trauma.

Limitations also occur on a microscopic and molecular level, as studies focused on understanding how fire affects the ability to generate genetic profiles suitable for forensic identification purposes, although still limited for human remains, suggest an acute and significant DNA degradation between 350 and 550 °C, and that the likelihood of generating high-quality mtDNA haplogroup calls decreases significantly at temperatures >550 °C [57].

Even bones and teeth, in fact, undergo a deep alteration of their macroscopic and microscopic structure, so that the analysis of DNA traces and/or lesions becomes highly unreliable. Dental lesions, for example, are often limited to those affecting the supportive structure, since the tooth enamel tends to shatter during cremation and is frequently not recovered while joint diseases may be under-represented due to loss of the trabecular bone because of osteoporotic bone crumbling in cremation or preferential taphonomic destruction and surviving evidence for fractures and weapon trauma is relatively rare.

Accordingly, Harbeck et al. [58] highlighted how the genetic profile of cremation remains is highly unreliable and not suitable for forensic purposes (e.g., identification, paternity testing) as, similarly, Von Wurmb-Schwark et al. [59] showed how short tandem repeats typing obtained from the remains of a burned body provided no match of the alleles from genetic profiles taken prior to cremation, being subject to contamination during post-cremation processing (i.e., handling of the ashes, grinding of bones, etc.).

Keeping these significant issues in mind, the worldwide increase in cremation rates must be followed by the ability to undeniably identify a non-natural cause of death as well as to guarantee the availability of at least a small number of biological samples, otherwise permanently lost, for any forensic purpose.

To the best of our knowledge, at this time, no homogeneous international methodology capable of satisfying these criteria is to be found, as shown by the unresolved controversy regarding the need for a second external examination [21,24,25] and the doubts concerning the accuracy of medical certificates authorizing cremation [22,23].

Instead, different countries seem to give some common answers that, however, do not fully exclude the presence of all issues.

For example, the common practice of subordinating the cremation of a body to the inspection of a medical examiner makes the decision strictly affected by the preparation of the operator who, if not properly trained, might fail to identify a violent or non-natural cause of death.

On the other hand, the authorization for cremation expressed by the relatives of the deceased in the absence of his or her written will may override the wishes of the deceased not to have access to such a burial practice.

Significant differences also exist, with countries trying to tackle in unique ways some of the issues related to cremation.

German legislation, for example, tries to address the problem of the not always optimal experience of the medical examiner providing for a second medical examination. The idea behind this legislative provision can be found in the numerous deficiencies identified in the first medical examination [22,23], often carried out by non-specialized operators making it essential for the second examination to be carried out by a doctor who has been authorized to do so by the lower health authority or who is a member of this authority. Specifically, only physicians who are permitted to use the specialization “Forensic Medicine”, “Pathology” or “Public Health Care” or who belong to an institute specialized in forensic medicine or pathology may be authorized.

Yet, as seen, this approach is controversial, and in different countries, a single medical examination, often combined to a detailed study of early and late medical records of the dead, is considered exhaustive. Issues, however, may still arise in countries where medical records are not always rapidly available or in cases of no records present whatsoever.

The utmost importance of the preliminary step of a medical examination prior to cremation makes it advisable to proceed to additional international studies to precisely identify the characteristics and skills of the doctor who should carry out such activities and to evaluate if a single medical examination, especially if in absence of clinical records, should still be regarded as reliable.

On the other hand, a comprehensive study of the costs of a second medical examination could give a clearer view on the actual viability of this methodology on a global scale.

Another significant difference can be found in the Italian obligation for the medical examiner to collect samples of biological fluids and skin annexations from the body and store them for a minimum period of ten years.

The reason behind this provision of law in Italy can be found in the need to collect biological samples for future parentage testing requests; otherwise, it is permanently lost in cases of cremation.

With the necessary premise that problems with its application throughout the Italian territory are present to this day, this practice could undoubtedly allow retaining sufficient material for any forensic study for a total period of 10 years, with the loss of any possibility to have a macroscopic study of injuries and/or cut incisions of bones or body remnants still present, but the chance to perform toxicological and genetic exams is otherwise impossible.

This procedure would also be relevant for countries which routinely investigate non-natural deaths, significantly benefiting of the presence of sample collection before cremation, as if a cause of death was initially misinterpreted as natural and the body was then cremated, any medico-legal issues arising after a long time since death (e.g., paternity tests, toxicological and/or histological analyses in deaths initially considered as natural, etc.) would be practically unsolvable.

Finally, in cases of mass disasters [60], the opportunity to perform routine autopsies and/or forensic exams could be significantly reduced, making it essential to ensure in some way the presence of biological samples to be analyzed in the future, which are otherwise irretrievably lost.

In a climate of uncertainty around the accuracy of post-mortem examinations and certification detected in some countries, there is no doubt that the Italian approach could give such significant guarantees for future forensic issues with no doubts to be left in a world of increasing cremation rates.

Given this significant benefit, it is important to say that the Italian approach is challenging under several aspects: the need for a unified sample collection protocol, the need for an adequate structure for sample storage, the implications concerning privacy [61] and consent and, especially, the actual cost–benefits rate.

As for this hypothesis, high priority should be given to the definition of a single, international protocol regarding the type and method of collection and preservation of biological samples, custody times and costs of management as well as disposal procedures at the end of the 10-year period.

From this perspective, because of the issues related to the disappearance of any adequate future material for macroscopic exams still persist, it would be strongly advised for medical examinations to be carried out by a properly trained forensic medicine specialist or doctor of the Forensic Medicine Operative Units.

After excluding causes of death other than natural, following the request for cremation, the examiner would then proceed to precise identification of the body, fill out specific consent and privacy informative forms and then proceed to collect biological samples (e.g., the buccal mucosa, skin annexes and, in the event of a negative result, a blood sample and/or vitreous humor).

The production of an adequate consent form [62,63] for collection, custody and further analysis of biological samples from deceased subjects is as much of critical importance as it still is a vastly unexplored ground [64], which is only partially addressed with the premise that if it is known that the deceased did not hold any objection to the procedure, a presumption of altruistic intent could take place and, in this case, a next-of-kin could authorize the sampling [65].

The consent form, strictly compliant to the legislation of the country in which it is adopted, should be signed in the presence of witnesses, also identified, and it should adequately inform about the types of samples collected, their collection, their storing, any potential mode and purpose of using as well as their future disposal after ten years. Further studies are strongly recommended to correctly produce such consent form, as the ones produced by Local Health Authorities of Campania and Sardegna seem to be rather limited in their information contents, so that they cannot be considered fully satisfying. Specifically, Sardegna did not actually produce an informed consent form, while the one produced in Campania strictly limits to citing the Italian Law [26].

Alongside the consent form, an exhaustive privacy informative [66,67] would be needed, thoroughly illustrating the chain of custody of said samples (potentially built by the Local Health Authority Custody Officer) and the privacy implications in terms of future access, use and disposal. For this matter, the consent forms of Sardegna and Campania do not cite in any way privacy matters, making it essential to initiate specific studies aiming at the production of an adequate privacy informative, trying to address the challenges connected to the limited knowledge in terms of deceased privacy.

As for the question of which are the best samples to collect, the Campania proposal seems to be the better choice: collecting buccal mucosa and skin annexes (e.g., hair) is a non-invasive and cost-effective procedure, the samples are easy to store and are an optimal, well-known source for future genetic and toxicological exams [68,69,70,71].

The proposal of Sardegna, instead, seems to carry higher costs, as formalin fixation should be guaranteed for every single procedure, even thought it would guarantee fixated skin flaps, so biological samples of higher quality for future histological evaluation. On the other hand, toxicological exams could be difficult if not impossible to perform, and genetic evaluations could be influenced by possible DNA degradation. However, the removal of pacemakers provided by Sardinia protocol is not present in the one produced by Campania and should be universally adopted.

All documentation relating to the identification of the deceased and witnesses should then be handed over, together with the samples, to a Local Health Authority Custody Officer that manages its storage, access and future disposal after 10 years. The guidelines produced in Campania seem to be more detailed than the ones produced in Sardegna, which do not specify any form of chain of custody, so that they could be adopted almost in their entirety.

As for the costs of said procedures, of likely medium-high entity, this matter should be approached keeping in mind that the impact on some, otherwise unsolvable, forensic cases should be considered priceless on both a macroscopic scale (public security) and a microscopic scale (e.g., paternity tests, urn content identification etc.). Campania and Sardegna, for this matter, try to mitigate the costs partially charging the citizens who request cremation, making it essential, once again, to proceed with further discussions to assess the sustainability of said procedures both at a state and citizen level.

## 5. Conclusions

A lot of similarities, but also some significant differences, were found in the comparative study of international legislation of cremation.

Parallels were identified in the need for a certificate that excludes any medico-legal issues and non-natural causes of death and in the consent to cremate a body relying on the deceased person that had expressed during his or her lifetime the will to be cremated or onto family members who, after death, will declare this wish.

On the other hand, significant differences were found in the German and Italian approach.

German law provides the need for a second medical examination carried out by a specialized doctor to guarantee an accurate evaluation of any suspect violent death.

Italian law, instead, provides the obligation for the medical examiner to collect samples of biological fluids and skin annexations from the body and store them for a minimum period of ten years.

As illustrated, a common response to the medico-legal issues of cremation usually consists in the total trust in external examinations together, if possible, with medical records of the deceased. However, controversy still exist around this methodology, as a lot of issues remain unsolved.

Although not fully applied in Italy itself and despite limits related to its inhomogeneous application throughout the national territory, the Italian approach is a unicum within international legislation in theme of cremation that gives a unique approach and a concrete plausible solution to a lot of the medico-legal issues raised by this practice, allowing the presence of a substrate of priceless value for genetic and toxicological exams during at least ten years following death.

The proposal of the Campania region, with the addition of the pacemaker removal guidelines of Sardegna, seems to be adequate on different levels. Still, it is imperative to improve on some identified deficiencies and look deeply into its privacy and consent implications, cost-benefits rate, sample collection and storage protocol.

## Figures and Tables

**Table 1:** Main similarities and differences among national cremation laws.

Country	Request Submission	Second Post-Mortem Medical Examination	Sample Collection
Italy	Deceased person will to be cremated expressed in writing by family members	Not provided	Biological fluids and skin annexations from the body stored for ten years
United Kingdom	An executor of the deceased person or a near relative who has attained the age of 16	Not provided	None
France	Written expression of the last wishes of the deceased or a request of any person who is qualified to provide for the funeral and provides proof of his marital status and domicile	Not provided	None
Germany (Berlin)	Written declaration of intent from the deceased or the next of kin	Carried out by a doctor who has a specialization in “Forensic Medicine”, “Pathology” or “Public Health Care” or who belongs to an institute specializing in forensic medicine or pathology	None
Spain (Castilla y Leon)	Document of last wills where the interested person or a first line relative certifies the wish of that person to be cremated.	Not provided	None
Portugal	Requested while still alive, exposing the individual’s will and the clarity of not having any pending issues with justice, or, after death, the request must be made by the executor in compliance with a testamentary provision, the surviving spouse, the person who lived with the deceased in conditions similar to those of the spouses, any heir, any relative [42].	Not provided	None
Norway	Made by the person who arranges for the deceased’s funeral.	Not provided	None
United States (New York State)	Made by the legal next of kin.	Not provided	None
United States (Florida)	Made by the legally authorized person contracting for cremation services which designates her or his intentions in a signed declaration of intent.	Not provided	None
Japan	Made by the person who arranges for the deceased’s funeral.	Not provided	None

**Table 2:** Main medico-legal issues related to cremation.

Main Medico-Legal Issues Related to Cremation
Correct ashes identification and reconduction to a body. No valid substrate for any future histological, toxicological or genetic exam.
No element to trace the causes and time of death.
Disappearance of any lesions regarding soft tissues or bones.
Risk of contaminations of biological samples during post-cremation processing.
Profound macroscopic and microscopic distortion of bones’ structure.
Ambiguity in terms of consent in absence of an explicit and written will.

## Data Availability

These data can be found here: [https://www.funerali.org/wp-content/uploads/File/Statistiche/Italia/SEFIT_Stat2021.pdf (accessed on 15 October 2022); https://www.gazzettaufficiale.it/eli/id/2001/04/19/001G0183/sg#:~:text=La%20presente%20legge%20discplina%20la,%2C%20ai%20sensi%20dell’art (accessed on 15 October 2022); https://www.legislation.gov.uk/uksi/2008/2841/contents; https://gesetze.berlin.de/bsbe/document/jlr-BestattGBErahmen (accessed on 15 October 2022); https://www.legifrance.gouv.fr/codes/article_lc/LEGIARTI000042661730/ (accessed on 15 October 2022); https://www.saludcastillayleon.es/es/sanidad-mortuoria/legislacion-sanidad-mortuoria; https://dre.pt/dre/detalhe/decreto-lei/411-1998-286106 (accessed on 15 October 2022); https://lovdata.no/dokument/NL/lov/1996-06-07-32/KAPITTEL_4#%C2%A723 (accessed on 15 October 2022); https://www.nysenate.gov/legislation/laws/PBH/4140 (accessed on 15 October 2022); https://www.cdc.gov/phlp/publications/coroner/florida.html (accessed on 15 October 2022); https://www.mhlw.go.jp/bunya/kenkou/seikatsu-eisei15/ (accessed on 15 November 2022), https://www.oecd.org/sti/emerging-tech/guidelines-for-human-biobanks-and-genetic-research-databases.htm (accessed on 27 September 2022)].
